# Prebiotics for Lactose Intolerance: Variability in Galacto-Oligosaccharide Utilization by Intestinal *Lactobacillus rhamnosus*

**DOI:** 10.3390/nu10101517

**Published:** 2018-10-16

**Authors:** Jason W. Arnold, Joshua B. Simpson, Jeffery Roach, Jose M. Bruno-Barcena, M. Andrea Azcarate-Peril

**Affiliations:** 1 Center for Gastrointestinal Biology and Disease, Division of Gastroenterology and Hepatology, and UNC Microbiome Core, Department of Medicine, School of Medicine, University of North Carolina, Chapel Hill, NC 27599, USA; jason_arnold@med.unc.edu (J.W.A.); joshsimp@live.unc.edu (J.B.S.); 2 Research Computing, University of North Carolina, Chapel Hill, NC 27599, USA; jeff_roach@unc.edu; 3 Department of Plant and Microbial Biology, North Carolina State University, Raleigh, NC 27607, USA; jbbarcen@ncsu.edu

**Keywords:** prebiotics for lactose intolerance, galacto-oligosaccharides, *Lactobacillus*, probiotics, β-galactosidases

## Abstract

Lactose intolerance, characterized by a decrease in host lactase expression, affects approximately 75% of the world population. Galacto-oligosaccharides (GOS) are prebiotics that have been shown to alleviate symptoms of lactose intolerance and to modulate the intestinal microbiota, promoting the growth of beneficial microorganisms. We hypothesized that mechanisms of GOS utilization by intestinal bacteria are variable, impacting efficacy and response, with differences occurring at the strain level. This study aimed to determine the mechanisms by which human-derived *Lactobacillus rhamnosus* strains metabolize GOS. Genomic comparisons between strains revealed differences in carbohydrate utilization components, including transporters, enzymes for degradation, and transcriptional regulation, despite a high overall sequence identity (>95%) between strains. Physiological and transcriptomics analyses showed distinct differences in carbohydrate metabolism profiles and GOS utilization between strains. A putative operon responsible for GOS utilization was identified and characterized by genetic disruption of the 6-phospho-β-galactosidase, which had a critical role in GOS utilization. Our findings highlight the importance of strain-specific bacterial metabolism in the selection of probiotics and synbiotics to alleviate symptoms of gastrointestinal disorders including lactose intolerance.

## 1. Introduction

Lactose intolerance occurs as a result of decreased expression of intestinal lactase, and is often associated with lactose-induced abdominal pain, diarrhea, and intestinal distension [1]. Prebiotics are functional foods that stimulate the growth of gut native beneficial bacteria. Pure prebiotic galacto-oligosaccharides (GOS) have been shown to decrease symptoms of lactose intolerance [2,3,4,5]. GOS induced changes in the gut microbiota, increasing the abundance of lactose-fermenting *Bifidobacterium*, *Faecalibacterium*, *Lactobacillus*, and *Roseburia* species [3]. Changes to microbial diversity and microbiota composition suggest that members of the gut microbiota can revert lactose intolerance; however, the mechanisms by which GOS plays a role in this interaction have yet to be determined. Moreover, dietary GOS supplementation specifically promoted growth of bifidobacteria in humans, but this response was variable between individuals [3,6].

Commercially available GOS formulations are currently limited in their purity, with some having oligosaccharide contents as low as 48% weight/volume. Commercial formulations often contain some fraction of glucose (0–22%), galactose (0–39%), and lactose (0–23%) [7]. The high concentration of lactose in the GOS preparation is of particular concern when the supplement will be administered to lactose-intolerant individuals. In clinical interventions aimed to determine the efficacy, safety, and tolerability of pure GOS in subjects with moderate to severe lactose intolerance [3,8] the prebiotic was administered as a powder (5 to 7.5 grams twice daily). Likewise, the chemical formulation of prebiotics directly impacts microbial fermentation and growth at the strain level. For example, *L. plantarum* strain CLC17 is able to grow in a variety of GOS formulations including 4′-galactosyl-lactose, 6′-galactosyl-lactose, and pure lactose, while *L. plantarum* strain CLB7 is unable to utilize lactose or 4′-galactosyl-lactose [9], highlighting the importance of the prebiotic formulation in the microbial physiological response. Similarly, non-GOS components of dietary prebiotic formulations can impact the composition of the gut microbiota. Lactose promotes the growth of lactic acid bacteria [10], galactose can be utilized by *Bacteroides* [11], and dietary glucose is utilized by most bacteria, increasing the abundance of Proteobacteria and reducing Bacteroidetes when fed in high concentrations [12].

Bacterial members of the human gut microbiota provide a myriad of health benefits, including immunomodulation [13,14], protection from pathogen colonization [15,16], and unique metabolic capabilities [17,18]. The complex relationships between microorganisms and their host have profound impacts on overall health, with dysbioses often associated with gastrointestinal diseases. Thus, modulation of the gut microbiota is emerging as an effective method to prevent and treat gastrointestinal disorders [19,20]. Methods for microbiota modulation include treatment with probiotics, which add exogenous beneficial bacteria to the community [21,22], and prebiotics, which promote the growth of beneficial endogenous microorganisms [23].

Probiotics are live microbes, which when administered in adequate amounts provide a benefit to their host [24]. Members of the genus *Lactobacillus* are commonly used as probiotics. Species and strains of this genus have been shown to modulate host gene expression [25], prevent pathogen colonization [16,26,27], modulate the immune system [28], and improve the metabolic potential of host and community through fermentation of complex carbohydrates [29]. The impact that probiotics have on their host is directly related to their physiology, often highly variable between strains. In order for a microorganism to act as a probiotic, it must first survive the stresses associated with the gastrointestinal tract [30], a capability that is highly variable between strains [31,32,33]. Similarly, the mechanisms by which probiotics provide a benefit vary between strains, including carbohydrate fermentation [9], an important feature of probiotics [34].

Research studies have determined that the majority of the intestinal diversity is present at the species and strain levels [33,35,36]. In the gastrointestinal tract, GOS are metabolized by bacterial β-galactosidases and β-glucosidases, though mechanisms for GOS transport and utilization vary between species and strains [37]. In this study, we tested a high-purity formulation to yield results with a reduced or complete elimination of biases introduced by non-GOS components. Our study aimed to characterize the molecular mechanisms by which pure GOS are metabolized by *L. rhamnosus* [38]. We included strains AMC143 and AMC010, isolated from healthy human infants [38,39], LGG, a well characterized probiotic, isolated from a healthy human adult [40], and Lc705, derived from dairy products [41]. These strains exhibited physiological differences [33] despite sharing nearly 97% nucleotide identity between strains [38]. We hypothesized that the origin of the strains would impact their ability to metabolize prebiotics. Therefore, strain variability could directly impact efficacy of prebiotics when utilized to prevent or treat symptoms of lactose intolerance.

## 2. Materials and Methods

### 2.1. Bacterial Strains, Culture Media, and Cultivation

*L. rhamnosus* strains used in this study are listed in Table 1. Strains were routinely propagated in MRS broth (Pronadisa, Madrid, Spain) at 37 °C without agitation, or on MRS agar plates containing 1.5% agar. For growth assays, MRS without dextrose (Pronadisa, Madrid, Spain) was supplemented with either 1% glucose, 1% lactose, 1% GOS, 1% cellobiose, or 0.1% lactose. The high-purity prebiotics used in the study were composed of GOS (90%) and lactose (10%) [42,43]. Since the GOS formulation contains 10% lactose, we used this disaccharide at 0.1% concentration as control for experiments using 1% GOS as the experimental condition. 

For growth assays, *L. rhamnosus* strains were cultured statically for 16 h in MRS broth at 37 °C. Freshly harvested cells were washed with MRS without dextrose and diluted 1:100 in MRS without dextrose supplemented with experimental carbohydrate sources. A total of 200 μL of bacterial suspensions were transferred to 96 well plates, sealed, and placed into a Tecan Infinite 200 Pro spectrophotometer (Tecan, Mendendorf, Switzerland). Measurements of optical density at 600 nm (OD_600nm_) were taken every 15 min during 24 h. Growth curves were plotted in Origin2016 (OriginLab, Northampton, MA, USA), and maximum specific growth rates (μ_max_) were calculated for each treatment. Each experiment was performed with eight biological replicates and three technical replicates.

### 2.2. Carbohydrate Fermentation Assays

*L. rhamnosus* strains were grown overnight in MRS growth media at 37 °C without agitation in duplicate. A total of 100 μL of cultures were harvested via centrifugation, and cells were washed twice with API^®^50CHL assay medium (BioMérieux, Marcy-Star, France). Washed cells were re-suspended in 10 mL of API^®^50CHL assay medium. Cell suspensions were then transferred to API^®^50CH and incubated at 37 °C for 48 h, as outlined by the manufacturer’s instructions. Carbohydrate fermentation was determined for each sample at 24 h and 48 h.

### 2.3. Determination of GOS Metabolites

*L. rhamnosus* strains were grown in minimal media (10 g yeast extract, 0.5 g (NH_4_)_2_HPO_4_, 0.05 g MnSO_4_, 0.005 g MgSO_4_·7H_2_O in 1 L H_2_O) supplemented with either 1% glucose or 1% GOS without agitation for 24 h at 37 °C. Samples were collected at early log growth phase (OD_600nm_ = ~0.3), late log growth phase (OD_600nm_ = ~0.7) and stationary phase and centrifuged to separate bacteria from media. The supernatants were filtered through 0.2 μM filter, flash frozen, and stored at −80 °C prior to High-Performance Liquid Chromatography (HPLC) analysis.

Products and substrates from each reaction were analyzed by HPLC (Shimadzu Corporation, Kyoto, Japan) under isocratic conditions at 65 °C and at a 0.5-mL·min^−1^ flow rate. The mobile phase was water using a Supelcogel Ca (300 mm by 7.8 mm, Supelco Analytical, Bellefonte, PA, USA) coupled to a refractive-index and ELSD detectors. The column was calibrated using the GOS species pentasaccharide, tetrasaccharide (purified in house), galactosyl lactose (Carbosynth, Berkshire, UK), and lactose. Absolute values of glucose and galactose (Sigma-Aldrich, St. Louis, MO, USA) were also obtained by measuring known quantities to generate a standard curve.

### 2.4. RNA Isolation

Three biological replicates of strains AMC010, AMC143 and Lc705 were grown to late log phase in MRS containing 1% glucose (OD_600nm_ = ~0.7), 1% GOS (OD_600nm_ = ~0.3), or 0.1% lactose (vehicle control, harvested at the same time as GOS-treated samples) prior to harvesting. After reaching late log growth phase, cells were harvested via centrifugation, flash frozen in RNAlater (Thermo Fisher Scientific, Waltham, MA, USA), and stored at −80 °C. RNA isolation was performed using the Qiagen RNeasy PowerMicrobiome kit (Qiagen, Valencia, CA, USA) following the manufacturer’s instructions. RNA was eluted in 50 μL RNase free water and quantified using the 2200TapeStation (Agilent Technologies, Santa Clara, CA, USA).

### 2.5. mRNA Sequencing

Depletion of Ribosomal RNA was carried out using the Ribo-Zero Gold Bacterial rRNA Removal Reagent (Epidemiology Kit) (Illumina, San Diego, CA, USA) according to the manufacturer’s instructions. Briefly, the rRNA-specific magnetic beads were removed from storage buffer and mixed with 500 ng of total sample RNA. Subsequently, rRNA removal solution was added and samples were incubated for 10 min at 65 °C. Finally, samples were placed on a magnetic stand for 15 min at 22 °C and mRNA was removed and immediately processed with TruSeq Stranded mRNA HT kit (Illumina, San Diego, CA, USA). RNA was mixed with Fragment-Prime mix and incubated at 94 °C for 8 min. Samples were immediately subject to first strand and second strand cDNA synthesis reactions, respectively, followed by 3′ end repair, adenylation and adapter ligation. After adapter ligation, the libraries were enriched by PCR using the following thermal cycling conditions: 98 °C for 30 s followed by 15 cycles of 98 °C for 10 s, 60 °C for 30 s and 72 °C for 30 s. A final extension step of 70 °C for 5 m was carried out following the last cycle. After enrichment, libraries were purified with magnetic beads (Beckman Coulter, Brea, CA, USA), washed with 80% ethanol and eluted in Tris pH 8.5. Following enrichment, cDNA was barcoded for multiplexing via PCR, using dual-index barcodes (index 1(i7) and index 2(i5)) (Illumina, San Diego, CA, USA) in a combination unique to each sample. Final ds cDNA was purified with magnetic beads. Library concentrations and quality were measured via TapeStation2200 (Agilent Technologies, Santa Clara, CA, USA). Barcoded libraries were pooled at equimolar concentrations and sequenced on the Illumina HiSeq platform (Illumina, San Diego, CA, USA).

### 2.6. mRNA Sequencing Data Analysis

Sequencing output from the Illumina HiSeq platform was converted to FASTQ format and demultiplexed using Illumina BclFastq 2.18.0.12 (Illumina, Santa Clara, CA, USA). Quality control was performed via FastQC on both raw and processed sequencing reads (Babrahm Institute, Cambridge, UK). Demultiplexed FASTQ sequence files were uploaded to Geneious software (Biomatters, Auckland, New Zealand). Sequencing reads from each treatment were mapped against Lc705 genome in Geneious software using “Geneious for RNA Seq” mapper, a minimum mapping quality of 30 (99.9% confidence), allowing gaps with a maximum of 10% per read, 20% maximum mismatches per read, with word length of 20 bases. Expression levels were calculated in Geneious and compared between treatment types. Genes identified as significantly differentially regulated between treatments via Binomial Distribution analysis in Geneious software and post-analysis Bonferroni correction for multiple comparisons (*p* ≤ 0.05) were further filtered to include only genes with 2-fold expression differences. Genes identified as significantly differentially regulated at ≥2-fold in at least one treatment type for each isolate were plotted as heat maps in OriginLab software (Origin Lab, Northampton, MA, USA), and compared between strains to show differences.

### 2.7. Generation of GOS Operon Insertion Mutant Strains

Electrocompetent *L. rhamnosus* cells were generated using a combination of previously described methods [44,45], with minor adjustments [33]. Insertional inactivation of the 6-phospho-β-galactosidase gene in *L. rhamnosus* AMC143 was performed as previously described [33,44]. Briefly, primers GOS_pβgal-F-EcoRI (5′-AATAGAATTCACAATTAGGCATTGCATTGCC-3′) and GOS_pβgal-R-BamHI (5′-TAATGGATCCTGAATCGAAGTGATGCAGCG-3′) were designed to amplify a 440 bp region of the GOS-specific 6-phospho-β-galactosidase gene in AMC143 (*p-βgal_lac3*). These primers included *EcoR1* or *BamHI* restriction sites for cloning into the pFAJ-5301 vector [13]. The resulting vector (pFAJ-pβGAL_*lac*3) was used to transform AMC143 by electroporation. Electrotransformed AMC143 cells were allowed to recover for 24 h in MRS at 37 °C without agitation, and subsequently plated on MRS containing erythromycin at a final concentration of 2 μg/mL. Individual erythromycin resistant colonies were selected after 48 h of growth at 37 °C and sub-cultured in MRS containing 2 μg/mL erythromycin overnight without agitation at 37 °C. Insertion mutant AMC143::*pβgal*_*lac*3 was verified by PCR amplification of the entire 6-phospho-β-galactosidase gene, using the primer set: V-pβGAL-F (5′-ATGAGTATGAACGACTGGCG-3′) and V-pβGAL-R (5′-TAATGCAAAAACAGACACGC-3′). Successful insertion mutants contained a 2 kb insertion in the target gene.

## 3. Results

### 3.1. Comparative Genomic Analysis of GOS Utilization Genes

**Figure 1:**
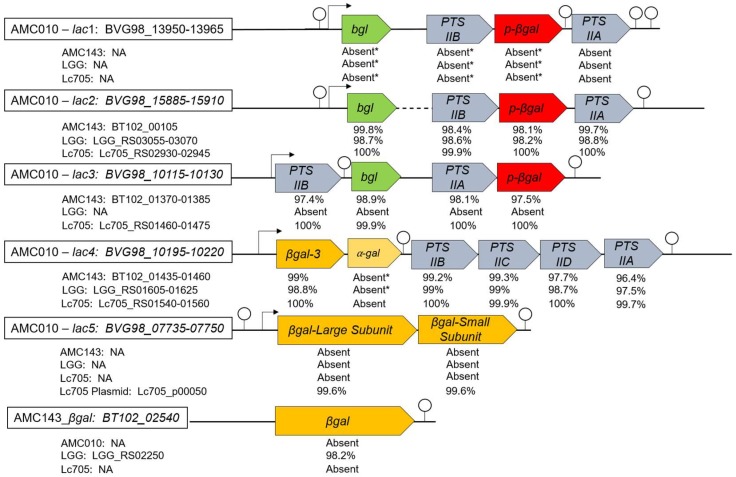
Comparative genomic of lactose metabolism operons in *Lactobacillus rhamnosus*. Genomes were analyzed using Geneious software, comparing operon organization and nucleotide identity between the human isolates AMC010, AMC143, LGG, and the dairy isolate Lc705. Promoters were identified using the BPROM (Softberry, Bangkok, Thailand) promoter prediction tool [48]. Terminators were identified using the ARNold terminator prediction tool [49]. * Genes showed >60% identity to homologs in lac2 operon; bgl: beta glucoside antiterminator; PTS: phosphotransferase system; NA: not applicable.

In *Bifidobacterium* and *Lactobacillus* species, GOS and lactose are metabolized by β-galactosidases and β-glucosidases [46]. A comparison of strains in this study showed that the genome of *L. rhamnosus* AMC010 had the highest number of genes annotated as ‘β-galactosidase’ or ‘phospho-β-galactosidase’. The genes were encoded in five different operons, each containing a different β-galactosidase gene (three different 6-phospho-β-galactosidases (EC 3.2.1.85), one β-galactosidase-3 gene, and β-galactosidase large and small subunit (EC 3.2.1.23)) (Figure 1). Operon AMC010_*lac*1 had no homologs in the other three strains evaluated in this study with a nucleotide identity below 70% when compared to the shared genes in the *lac2* operon of each of the other strains. This operon showed the highest identity with plasmids from *L. paracasei* and *L. rhamnosus* Lc705 (99% nucleotide identity). AMC010_*lac*1 shared a similar organizational structure with AMC010_*lac*2, but their identity was below 70%. Homologs to each gene in the operon AMC010_*lac*2 were identified in all four strains with 98% or higher nucleotide identity. Operon AMC010_*lac*3 was present in AMC143 and Lc705 but absent in LGG, and had a different organization with a putative terminator identified between the phosphotransferase system (PTS) subunit IIB and the *bgl* anti-terminator gene. AMC010_*lac*4 also contained a unique α-galactosidase gene absent in the other strains in our study. All of the other genetic components had homologs in the analyzed strains. The AMC010_*lac*5 operon contained large and small subunits of the β-galactosidase, and was absent in the genomes of the other strains in this study. However, this β-galactosidase_*lac5* gene was 99.6% identical to a β-galactosidase gene identified in Lc705 plasmid FM179324.1 [41], and 99% identical to a chromosomally encoded β-galactosidase in *L. plantarum* strain HFC8 [47], suggesting that this operon may be plasmid encoded in AMC010. Finally, AMC143 encoded a unique β-galactosidase gene absent from Lc705 and AMC010, but 99% identical to other *L. rhamnosus* strains. Operons *lac2*, *lac3*, and *lac4* were highly conserved in *L. rhamnosus*, but absent in other *Lactobacillus* species, with nucleotide identity below 30% in *L. casei*, a close relative to *L. rhamnosus*.

Analysis of potential transporters in the genome sequences of *L. rhamnosus* strains showed that AMC143 encoded the highest number of putative transporters of the phosphotransferase system (PTS) (99 genes), providing the strain with the capability to transport at least 16 different carbohydrate substrates based on our genome annotations (Table 2). Lc705 encoded 92 PTS genes, allowing for the transport of 17 unique carbohydrate substrates, the largest number among strains in this study. AMC010 encoded 92 PTS genes targeting 16 different substrates, and LGG encoded 78 transporters targeting 15 different substrates (Table 2). In addition to PTS transporters, *L. rhamnosus* strains encoded ATP-binding cassette (ABC) transport systems. These systems generally facilitate the transport of peptides and metals into the cell, but carbohydrate-specific ABC transporters including transporters for *N*-acetyl-d-glucosamine and maltose/maltodextrin were identified, and were highly conserved between strains, with nucleotide identities over 98%. Few ABC transport genes have been characterized in *L. rhamnosus*. Genome analysis revealed that AMC010 encoded 127 putative ABC transport genes, AMC143 encoded 133 ABC transport genes, LGG encoded 196, and Lc705 encoded 215, many of which were uncharacterized, with unknown substrates. In *L. acidophilus*, the *lacS* permease is responsible for the internalization of GOS [37]. Our analysis revealed that this gene was absent in *L. rhamnosus*, suggesting a different mechanism for GOS uptake.

Galactose is imported by the galactokinase *galKETRM* operon in *L. casei* strain 64H [50]. A homolog of this operon was identified in *L. rhamnosus* (designated as *galKETRM*), though only conserved with 60% nucleotide identity compared to *L. casei* 64H. However, a second galactose metabolism operon (designated as *galKETRMa* in this study), distinctly different from *L. casei galKETRM* operon, was present in the four *L. rhamnosus* strains with 98.5% nucleotide identity between strains. Our genomic analysis regarding conserved and variable carbohydrate transport and metabolism systems suggested that variability in the ability of a microorganism to utilize carbohydrates occurs at the strain-level.

### 3.2. Carbohydrate Utilization by L. rhamnosus

The genomic differences identified between strains led us to assess their impact on phenotype. Carbohydrate fermentation profiles generated using the API CH50 system showed unique profiles for each strain (Supplemental Table S1). The probiotic strain LGG was unable to ferment lactose, while all other strains showed positive signals for lactose fermentation within 24 h. Strains Lc705 and AMC143 were the most efficient at fermenting GOS, fully fermenting the substrate within 24 h, while AMC010 only partially fermented GOS. LGG was also unable to ferment GOS at 24 h; however, all strains exhibited at least partial utilization after 48 h. The fermentation of other carbohydrates including gentiobiose, maltose, amygdalin, sorbitol, sorbose, rhamnose, and ribose, varied between strains, revealing unique carbohydrate fermentation profiles for each strain. L-fucose, a major component of intestinal mucus, was fermented by neither AMC010 nor Lc705, but was utilized by LGG and AMC143.

**Figure 2:**
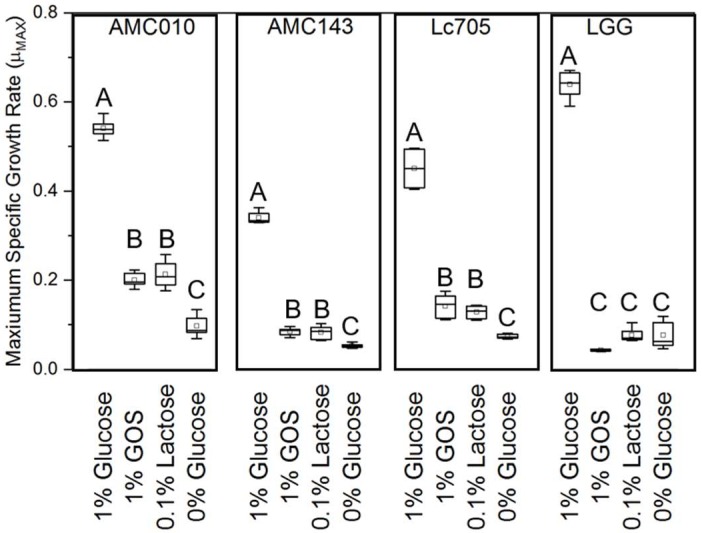
Specific growth rates of *L. rhamnosus* strains. Growth rates were calculated for each strain in MRS containing 1% glucose, 1% GOS, 0.1% lactose (the lactose component of GOS formulation), and in MRS without carbon and energy sources. Eight biological replicates, each in triplicate (three technical replications) were included in each experiment. Letters (A, B, C) represent statistical differences between treatments in each strain (*p* < 0.05).

Culture experiments in a complex medium containing 1% glucose, 1% GOS or 0.1% lactose (*w*/*v*) as a sole carbohydrate source showed that LGG had the highest maximum specific growth rate in glucose (0.68 h^−1^) but was unable to grow in GOS or lactose; consequently, it was omitted from subsequent expression experiments. Glucose was also the preferred substrate for AMC010, but the difference in specific growth rate between GOS and lactose was minimal (0.19 h^−1^ and 0.22 h^−1^, respectively). Lc705 was able to utilize both GOS and lactose, growing at approximately the same rate in each (0.14 h^−1^ and 0.11 h^−1^, respectively) (Figure 2). AMC143 exhibited the lowest specific growth rates in this study (0.34 h^−1^ in glucose and 0.08 h^−1^ in GOS and lactose) (Figure 2).

**Figure 3:**
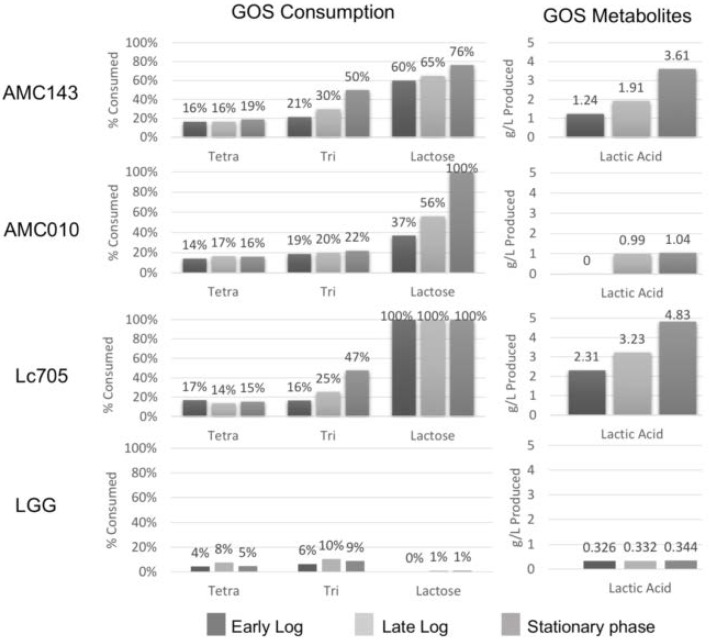
GOS utilization and generation of secondary metabolites. Minimal media containing 1% GOS as a sole carbohydrate source were analyzed by HPLC to determine residual carbohydrates after incubation with each strain to early logarithm, late logarithm, and stationary growth phase. Three biological replicates, each in triplicate (three technical replications) were included in each experiment. The utilization of lactose and GOS varied between strains, and the production of lactic acid correlated with utilization.

Analysis of GOS fermentation products showed that AMC010 fully metabolized lactose by the time it reached stationary phase 24 h post inoculation, while leaving the majority of GOS tri- and tetrasaccharides intact, confirming our previous observation that AMC010 utilized primarily the lactose component of the GOS formulation. Conversely, AMC143 simultaneously fermented lactose and the trisaccharide, only utilizing larger GOS oligomers when trisaccharide concentrations were reduced (Figure 3). Finally, Lc705 fully fermented the lactose in the medium by early log growth, and began the utilization of trisaccharides once lactose was no longer present. These sugar consumption patterns correlated with the generation of products, showing that both Lc705 and AMC143 generated over four times the amount of lactate (5 g/L and 4 g/L) than AMC010 (1 g/L) when grown in GOS.

### 3.3. Gene Expression Analysis of L. rhamnosus in Medium Containing GOS as a Carbon Source

**Figure 4:**
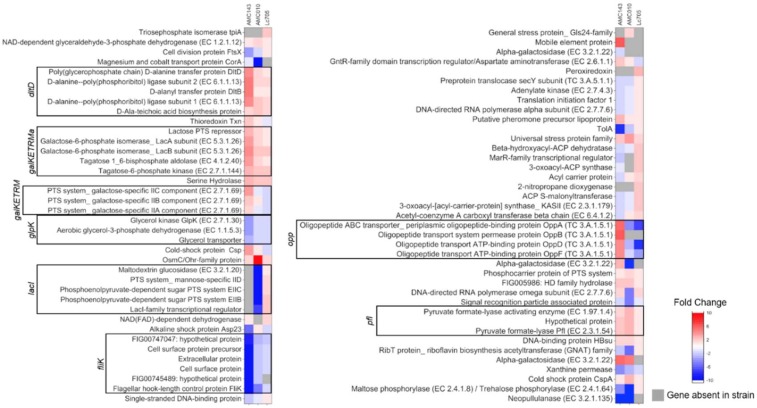
Transcriptomics analysis. Heat maps for AMC143, AMC010, and Lc705 were generated from sequencing reads mapped to their respective genome sequence and plotted to show fold changes between cultures in glucose compared to GOS. Boxed genes represent potential operons, and grayed genes represent genes absent in the associated strain. Three biological replicates were included in each experiment.

**Figure 5:**
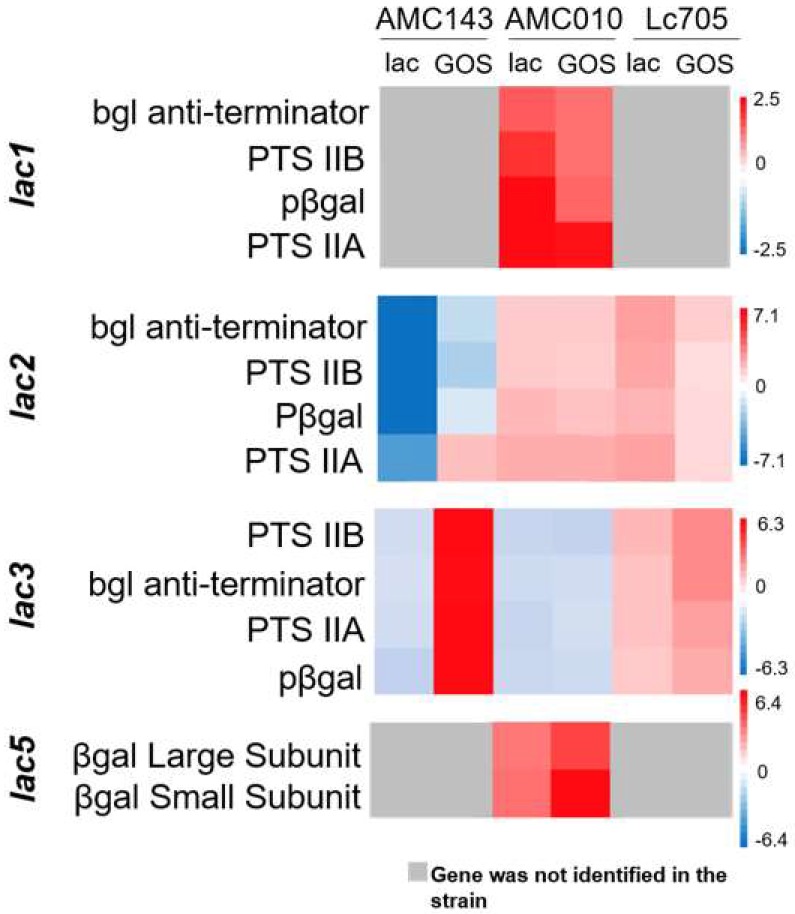
Expression of *lac* operons in GOS and lactose. Heat maps were generated from mRNA sequencing data for the identified *lac* operons in AMC143, AMC010, and Lc705, significantly differentially regulated by treatment with either GOS or lactose (*p* < 0.05 after Bonferroni correction). The fold change for each gene was determined by comparing expression data from lactose or GOS versus glucose. Gray boxes represent genes not identified in the corresponding genome. PTS: Phosphotransferase System.

In order to identify genes responding specifically to GOS, we compared the gene expression of *L. rhamnosus* strain cells harvested at mid-log phase (OD_600nm_ = 0.3–0.7) in MRS medium containing either 1% glucose, 1% GOS, or 0.1% lactose as sole carbohydrate sources (three biological replicates per treatment group). LGG was excluded from this analysis due to its inability to metabolize lactose and GOS. We identified 43 genes specifically overexpressed in response to GOS in AMC143, 16 genes in Lc705, and 21 genes in AMC010 when compared to expression levels in media containing glucose (Figure 4 and Supplementary Table S2). GOS treatment also resulted in underexpression of 31 genes in AMC143, five genes in Lc705, and 24 genes in AMC010 (Figure 4). The most overexpressed genes in AMC143 included the *lac3* operon, an oligopeptide ABC transport (*opp*) operon, and *L. rhamnosus*-specific galactose operon (*galKETRMa*). Additionally, we identified three hypothetical and uncharacterized genes (FIG00754012, FIG00751425, and FIG00744856) conserved in *L. rhamnosus*, *L. casei,* and *L. zeae,* overexpressed by over 4-fold in AMC143 when exposed to GOS (Figure 4 and Figure 5). When AMC010 was grown in GOS, overexpressed genes included the *lac5* operon β-galactosidase genes, *osmC*, a pyruvate formate-lyase operon, *galKETRMa* operon, and α-galactosidase genes. Interestingly, expression of the *lac3* operon was reduced in AMC010 exposed to GOS. Like AMC143, *lac3* was the most overexpressed operon when Lc705 was cultured in GOS-containing medium. Expression of an operon containing hypothetical cell surface proteins and a flagellar hook-length control gene (*fliK*) was reduced across all strains grown in GOS. Other genes differentially expressed in GOS are listed in Supplementary Table S2.

The *lac*3 operon was verexpressed 6-fold in AMC143 grown in GOS compared to glucose, but not in lactose (Figure 5). The same operon was overexpressed 3-fold in Lc705 grown in GOS but not in AMC010 cultured under either experimental condition. Operons *lac*1 and *lac*5 were both overexpressed in AMC010 grown in lactose or GOS, but were not differentially expressed in the other strains in this study. The *lac*2 operon was not differentially expressed in GOS or lactose in AMC010, but interestingly the homologous operon in AMC143 showed decreased expression in lactose-containing MRS (Figure 5). The highly conserved *lac4* operon (Figure 1) was neither over- nor underexpressed in media with GOS or lactose in any of the strains. Lactose-specific PTS transporters in *lac1*, *lac2*, and *lac3* operons were differentially regulated between strains exposed to GOS. Additionally, the expression of mannose-specific PTS transporters was down-regulated in AMC143 in GOS, but not in AMC010 under the same conditions. Galactose-specific PTS transporters were also up-regulated in AMC143 in GOS, but not in other strains.

With regards to transcriptional regulatory elements, the β-glucoside operon anti-terminator genes (*bgl* anti-terminator) present in operons *lac1, lac2*, and *lac3* showed the same expression trends as their corresponding operons (Figure 5). Additionally, GOS resulted in up-regulated expression of the lactose PTS repressor in AMC143, in accordance with repression of the *lac2* operon in this strain in GOS and lactose. In Lc705, a *lacI* family transcription repressor (LC705_RS01985), homologous to the LacI family DNA-binding transcriptional regulators in *L. casei* and *L. paracasei* (identified by BLASTx, Bethesda, MD, USA), was overexpressed in GOS and lactose but down-regulated in AMC010 grown under both conditions. This gene was absent in the genome of AMC143. Finally, exposure to GOS resulted in overexpression of an aspartate aminotransferase transcription regulator (*GntR*) and underexpression of a *marR*-family transcription regulator in AMC143. The *marR* gene was not differentially expressed in AMC010, but was over expressed by GOS in Lc705. Both genes were present in all *L. rhamnosus* strains, and highly conserved across *L. rhamnosus*, *L. casei*, and *L. paracasei*.

### 3.4. Functional Characterization of lac3, A GOS-Specific Operon of L. rhamnosus AMC143

**Figure 6:**
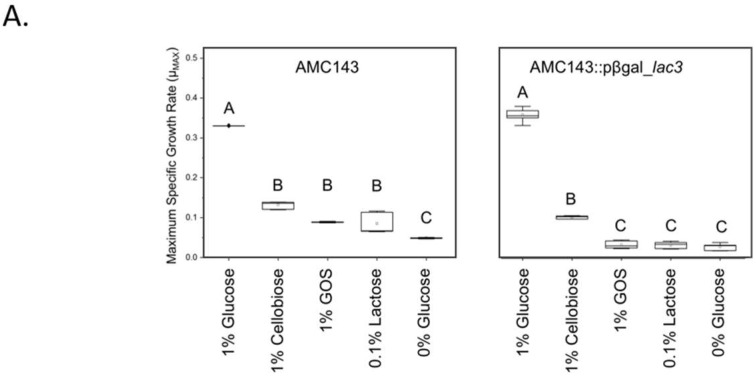

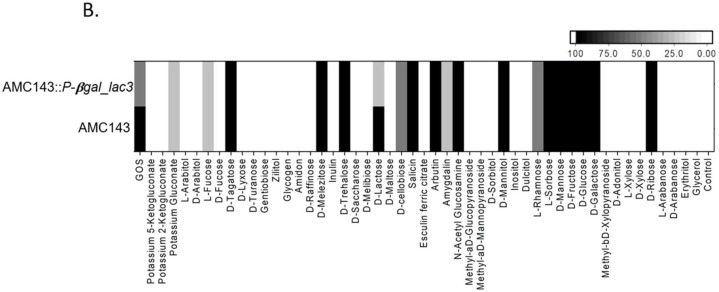
Growth and fermentation profiles of AMC143::*pβgal_lac3*. (**A**) Maximum specific growth rates for AMC143 and AMC143::*βgal_lac3* were determined in MRS containing 1% glucose, 1% GOS, 0.1% lactose, 1% cellobiose, and 0% glucose. Letters represent statistical differences between strain culture in each media type (*n* = 8, *p* < 0.05). (**B**) Carbohydrate utilization profiles were generated by API 50CH, revealing that the mutant was unable to ferment GOS and lactose after 24 h.

The 6-phospho-β-galactosidase (*p-βgal_lac3*) gene from the AMC143_*lac*3 operon was highly up-regulated in the presence of GOS, but not lactose. This operon was also overexpressed in Lc705, although to a lesser extent. To characterize the role of the 6-phospho-β-galactosidase gene and the *lac*3 in GOS metabolism, we inactivated the *p-βgal* gene by disrupting the coding region through site-directed insertional inactivation in AMC143, generating the AMC143::*p-βgal_lac3* strain. Inactivation of the *p-βgal_lac3* gene was confirmed by amplification and sequencing of the flanking regions in the section where the plasmid containing the gene fragment was inserted in the genome of AMC143 (not shown). The mutant strain was unable to grow in MRS broth containing either lactose or GOS as carbohydrate sources (Figure 6A). When grown in 1% cellobiose, both wild type and mutant strains grew at the same rate, confirming that the inactivated gene functions as a β-galactosidase and not a glucosidase (Figure S1). Carbohydrate fermentation profiles showed that AMC143::*p-βgal*_*lac3* was unable to utilize pure GOS and was deficient in its ability to ferment D-lactose at 24 h (Figure 6B). This data conflicted with transcriptomic profiles, which showed that *p-βgal_lac3* was not induced in AMC143 by exposure to lactose (Figure 5), suggesting a different mechanism for the internalization of lactose, despite the role of p-βgal_lac3 as the primary hydrolase for lactose fermentation.

## 4. Discussion

Pure galacto-oligosaccharides (GOS) have shown efficacy in alleviating symptoms associated with lactose intolerance [3,4]. However, variability in response to GOS has been reported [3,6]. Our previous work showed that the relative abundance of *Bifidobacterium* was inversely correlated with symptoms of pain and cramping in a Phase I clinical trial that evaluated pure GOS in lactose intolerant individuals [3], establishing a correlation between a measurable change in the gut microbiota, the ‘bifidogenic response’ (defined as an increase in abundance of this genus upon GOS consumption), and decreased lactose intolerance symptoms. These observations led us to hypothesize that the baseline composition of the gut microbiota at the strain level could contribute to response variability. We predicted that genomic and phenotypic differences at the strain level could impact GOS metabolism. In this study, we carried out an exhaustive characterization of the genetic systems involved in GOS metabolization in three strains of *L. rhamnosus* of intestinal origin, compared to a strain of dairy origin. The strains included in our analysis were a recognized human-derived probiotic strain (*L. rhamnosus* GG) [40], two strains previously isolated from infant stools (AMC143 and AMC010 [38,39]), and Lc705, of dairy origin [41]. We first performed comparative genomic analyses to identify operons encoding β-galactosidases and carbohydrate transport systems, finding that despite an overall identity between strains of over 95%, there were unique genetic components in each strain. Upon identifying these differences, we performed physiological assays to characterize the ability of each strain to utilize GOS and other carbohydrate sources, revealing significant variability between strains. Transcriptomic analyses, performed to identify genes that were differentially regulated in each strain when using GOS as a sole carbohydrate source, provided insight into the *lac*3 operon in AMC143, which was subsequently disrupted and identified as a critical component of GOS fermentation.

Genomic analyses of the strains used in this study revealed both unique (*lac1*) and highly conserved (*lac2, lac3*) PTS operons containing 6-phospho-β-galactosidase genes or other β-galactosidases (*lac4, lac5*). The operons present in all strains shared a high degree of nucleotide identity (96.4–100%). Similar operons containing 6-phospho-β-galactosidase genes have been described in other *Lactobacillus* species, including *L. gasseri*, *L. acidophilus*, and *L. casei* [51,52,53]. One of these hydrolases has been associated with the ability of *L. casei* strain BL23 to utilize oligosaccharides in human breast milk [54], similar to what we observed with AMC143*_pβgal_lac3*. Likewise, multiple 6-phospho-β-galactosidase enzymes have been described in *L. gasseri* strain JCM1031, *p-βgal 1* and *p-βgal 2*, each with unique metabolic capabilities and kinetics (116 μmol/mg/min and 86 μmol/mg/min, respectively) [55,56], suggesting that differential expression of hydrolase genes may play a role in bacterial physiology related to the utilization of carbohydrates. This finding corroborated earlier studies that identified multiple *p-βgal* genes in *L. gasseri* strain NCK334 [57].

Phosphotransferase systems (PTS) have been extensively characterized in *Lactobacillus* [54,58], including *L. rhamnosus* as a mechanism for the transport of tagatose [44] and other carbohydrates into the cytoplasm [59]. Although *L. casei*, a close relative to *L. rhamnosus*, was among the first species of *Lactobacillus* shown to utilize PTS transporters to incorporate GOS tri- and tetrasaccharides [54], GOS transport via PTS has not been reported in *L. rhamnosus*. The primary mechanism for GOS transport in other lactobacilli involves permeases, such as LacS in *L. acidophilus* [37] and RafP in *L. plantarum* [60], which are absent in the *L. rhamnosus* strains in this study. Our study showed GOS-specific overexpression of the PTS components IIA and IIB in the *lac3* operon in AMC 143 (BT102_01370, 01380); however, transport experiments will be essential to determine if these are involved in the incorporation of the oligosaccharides into the cell. Moreover, PTS transporters are highly specific for their substrates, but are often able to facilitate the transport of similar molecules [61]. As such, it stands to reason that lactose-specific PTS transporters may be capable of transporting larger GOS molecules into the cytoplasm due to their chemical similarity to lactose.

Our study showed that carbohydrate preferences varied between strains, and that this variability was a result of differential genetic capabilities and gene expression. No previous studies have shown the order of preference in which different oligosaccharides (di, tri, tetra or penta-saccharides) are consumed by different organisms, probably due to the lack of pure standard molecules for chromatography analysis,. Lc705, a strain of dairy origin, preferentially utilized lactose over trisaccharides, only metabolizing trisaccharides when lactose was not present, despite overexpression of the *lac3* operon in this strain when grown in GOS. This operon was found to be critical for GOS utilization by AMC143. Similarly, operon *lac2* was induced in both AMC010 and Lc705 by both lactose and GOS, but down-regulated in AMC143 under both conditions, suggesting a preferential mechanism for lactose utilization in these strains. Additionally, our data showed strains preferentially metabolized tri- over tetrasaccharides and longer oligosaccharides. We speculate that preference for trisaccharides is related with kinetics of GOS internalization, which has been shown to be important for the internalization of other carbohydrates [59,62].

Mobile genetic elements, including plasmids, allow for gene transfer and rapid evolution of bacterial populations [63,64]. Plasmids encoding enzymes relevant for carbohydrate metabolism have been described in *Lactobacillus* [52,65], suggesting that this group may have evolved to tolerate nutrient-limiting environments by carrying plasmids that extended their metabolic capabilities. Lc705 hosts plasmid FM179324.1 [41], which encodes a β-galactosidase gene 99.6% identical to the β-galactosidase gene in the AMC010 *lac5* operon. This plasmid-encoded β-galactosidase was not overexpressed in Lc705 grown in GOS or lactose compared to glucose, but had a higher expression level in AMC010 when exposed to either of those components in the media. This plasmid is absent in AMC143 and LGG (strains that grow less effectively in pure lactose), suggesting that the ability of Lc705 and AMC010 to utilize lactose may have evolved through horizontal gene transfer of this carbohydrate utilization pathway, a phenomenon previously described in *L. rhamnosus* [63].

Physiological assays and mRNA sequencing revealed that the utilization of GOS and lactose by AMC143 is controlled by a highly conserved “lactose-specific PTS system” (*lac*3 operon), also present in AMC010 and Lc705, but absent in LGG. The inability of LGG to utilize either lactose or GOS is likely a consequence of these 6-phospho-β-galactosidase and PTS transporter genes being absent from its genome. However, even the strains that do utilize both lactose and GOS do so in different ways. For example, Lc705 and AMC010 preferentially utilize lactose. Lc705 will only begin to metabolize GOS trisaccharides after lactose in its growth media has been fully expended, while AMC010 does not break down GOS trisaccharides well, even in the absence of lactose. AMC143, however, is able to utilize GOS trisaccharides, even while lactose is still present. These observations, combined with mRNA sequencing results, suggest that different systems for GOS fermentation are utilized in a strain-specific manner. Insertional inactivation of the *p-βgal_lac3* gene in AMC143 confirmed the role of this operon in GOS utilization. Marginal utilization of the prebiotic by the mutant strain suggested a secondary mechanism present for the metabolism of internalized GOS. We propose that either the PTS transporter genes in the *lac3* operon, or alternative transporters, still mediate GOS uptake in the mutant strain, where alternative, less-efficient enzymes metabolize the prebiotic, suggesting multiple mechanisms of utilization. This was reported in *Corynebacterium*, where carbohydrate transport occurs through both ABC and PTS transporters, allowing for more efficient transport than either system alone can provide [66]. Similar synergistic interactions between carbohydrate transport systems have not been described in *Lactobacillus*.

Gene expression varied between cells exposed to GOS and cells exposed to lactose compared to glucose, suggesting unique mechanisms for the utilization of each carbohydrate. In AMC143, inactivation of *pβgal_lac3* inhibited growth in lactose as well as in GOS, although growth in a medium with lactose did not result in overexpression of the *lac3* operon compared to glucose. Similarly, disruption of *AMC143_pβgal_lac3* did not affect growth rate in cellobiose, indicating that *pβgal_lac3* is indeed a β-galactosidase rather than a glucosidase. RNA sequencing showed overexpression of an operon containing cellobiose-specific PTS transporters and a 6-phospho-β-glucosidase gene in AMC143 (BT102_10960-10980) when exposed to lactose. This suggests that the cellobiose-specific PTS transporter in AMC143 may facilitate the transport of lactose, while a different system in AMC143 is used for the transport of GOS. Despite utilizing different transport systems, the same galactosidase (*pβgal_lac3*) is used to metabolize both carbohydrates.

Our data on strain-level variability strengthen the argument for exhaustive physiological characterization of individual probiotics, especially in the context of synbiotic development. A recent study showed that probiotic *Bifidobacterium* strains given to obese adult individuals along with GOS improved intestinal physiology, without synergistic effects. Both treatments improved colon permeability, however synergistic benefits were not shown in patients treated with both the probiotic *Bifidobacterium* and GOS together [67]. Since different bacterial strains can have different capabilities to utilize prebiotics, without an in-depth characterization of individual strains it is challenging to determine whether or not the lack of synergy in a synbiotic mixture is due to inability of the probiotic bacteria to ferment the prebiotic substrate. Likewise, it is important to note that bacterial physiology within a complex community most probably will not mirror responses from an in vitro system, as undefined or uncharacterized microorganisms will also contribute to GOS metabolism. Further studies, including validation experiments in complex gut-mimicking bioreactors, will be useful to determine if the strains perform in a similar manner within a complex gut ecosystem.

Our study highlights the importance of combining classical microbiology approaches with high-throughput sequencing techniques when studying microorganisms of probiotic potential. Whole Genome Shotgun (WGS) sequencing is widely used to identify prevalent genes and extrapolate function from data [68,69]; however, research demonstrates that, while the presence of a gene may provide a basis for a hypothesis regarding function, physiological and expression data are required to determine functionality.

## Figures and Tables

**Table 1:** Bacterial strains used in this study.

Strain	Origin	Reference
*L. rhamnosus* GG	Healthy human isolate	[41]
*L. rhamnosus Lc705*	Fermented dairy product	[41]
*L. rhamnosus AMC010*	Human infant stool	[38]
*L. rhamnosus AMC143*	Human infant stool	[38]
*L. rhamnosus AMC143::P-βgal_lac3*	This study	

**Table 2:** Quantification and qualification of annotated phosphotransferase system (PTS) transporters in *L. rhamnosus* strains. Open Reading Frames (ORF) numbers for each gene can be found in Table S2.

Strain	Carbohydrate Transported							
	α-Glucoside	Ascorbate	β-Glucoside	Cellobiose	Fructose	Galactitol	Galactosamine	Galactose
LC705	1	8	5	9	18	5	0	0
LGG	0	5	3	10	12	9	0	0
AMC143	0	0	5	24	8	3	4	6
AMC010	0	0	4	14	10	5	4	6
	Glucitol	Hyaluronate-Oligosaccharide	Uncharacterized	Glucose	Lactose	Maltose	Mannitol	Mannose
LC705	1	0	10	4	6	1	2	8
LGG	1	0	7	3	6	0	2	13
AMC143	4	2	7	0	5	2	4	15
AMC010	4	2	3	0	8	4	4	13
	Mannose/Fructose/Sorbose	*N*-acetyl galactosamine	*N*-acetyl glucosamine	Sorbitol	Sorbose	Trehalose	Sucrose	Total
LC705	2	3	3	2	3	1	0	46
LGG	1	0	0	2	2	1	1	39
AMC143	0	2	0	0	3	1	4	50
AMC010	0	2	0	0	3	2	4	43

## Supplementary Materials

The following materials are available online at http://www.mdpi.com/2072-6643/10/10/1517/s1. Figure S1: Growth of AMC143 and AMC143::*pβgal_lac3* in lactose and cellobiose. Growth curves were calculated for wild type and mutant AMC143 strains by measuring the optical density at 600 nm (OD_600nm_) every 15 min over a 24 h period in MRS media containing either 1% lactose or 1% cellobiose as a sole carbohydrate source (*n* = 8), Table S1: Carbohydrate utilization profiles of *L. rhamnosus*. The ability of AMC143, AMC010, Lc705 and LGG to ferment different carbohydrates was assessed using the API 50CH test kit. Two biological replicates were included in each test. The degree of utilization for each substrate is presented in the table, Table S2: Differential gene expression in GOS and lactose. Expression data from AMC143, AMC010, and Lc705 cultures in 1% GOS or 0.1% lactose (vehicle control), each run in triplicate, compared to glucose. The *p*-value was calculated by binomial distribution analysis in Geneious software with Bonferroni correction for multiple comparisons.
